# A review of combined imaging and therapeutic applications based on MNMs

**DOI:** 10.3389/fchem.2025.1595376

**Published:** 2025-05-26

**Authors:** Yiqing Yang, Peihong Teng, Shaonan Yu, Yuran Meng, Jinjie Zuo, He Guo, Guifeng Liu

**Affiliations:** ^1^ Department of Radiology, China-Japan Union Hospital of Jilin University, Changchun, Jilin, China; ^2^ Department of Radiation Oncology, China-Japan Union Hospital of Jilin University, Changchun, Jilin, China

**Keywords:** magnetic nanomaterials, multimodal imaging, cancer therapy, theranostics, personalized therapy

## Abstract

Magnetic nanomaterials (MNMs) are nanoscale materials with inherent magnetic properties that can respond to external magnetic fields, typically composed of magnetic metals or metal oxides. These materials exhibit broad application prospects in medical imaging, targeted drug delivery, and cancer therapy due to their exceptional magnetic properties, biocompatibility, and surface functionalization capabilities. As multifunctional imaging contrast agents, magnetic nanomaterials have been extensively employed in magnetic resonance imaging, computed tomography, and positron emission tomography to enhance multimodal imaging, thereby improving image resolution and diagnostic accuracy. Additionally, as targeted drug carriers, they can be guided by external magnetic fields to achieve precise drug delivery, enhancing therapeutic efficacy while minimizing systemic side effects. In therapeutic applications, magnetic nanomaterials have been utilized in magnetic hyperthermia therapy and photothermal therapy, where they generate localized heat via alternating magnetic fields or photothermal conversion effects, enabling tumor microenvironment modulation and precise tumor ablation. This review systematically summarizes recent advances in the use of MNMs for medical imaging and therapy, with a particular focus on key technical challenges and emerging opportunities to achieve synergistic imaging and therapeutic functions. This work aims to provide valuable insight into the development of MNMs for applications in precision medicine and personalized therapy.

## 1 Introduction

Over the past decade, magnetic nanomaterials have garnered significant attention in the biomedical field due to their unique physicochemical properties, leading to substantial advancements in their development (Shasha and Krishnan, 2021). Their applications in medical imaging are primarily based on magnetic responsiveness, which allows them to modulate the local magnetic environment under an external magnetic field, thereby significantly enhancing imaging contrast. Among these applications, magnetic resonance imaging (MRI) contrast agents are the most widely studied (Yan et al., 2023; Dadfar et al., 2019). Superparamagnetic iron oxide nanoparticles (SPIONs) function as T2 contrast agents by shortening the T2 relaxation time, increasing image contrast, and rendering pathological regions darker (Lin et al., 2015). In contrast, ${\text{Gd}}^{3+}$ -doped magnetic nanocomposites serve as T1 contrast agents, enhancing image brightness and improving the visualization of tissue structures (Ni et al., 2016). Furthermore, magnetic nanomaterials can be integrated with computed tomography (CT), positron emission tomography (PET), and single photon emission computed tomography (SPECT) by surface modification with high Z elements or radiolabeling, thereby enabling multimodal imaging and improving the accuracy of both anatomical and functional imaging (Chen et al., 2021; Accogli et al., 2021). In particular, magnetic nanomaterials can also be conjugated with fluorescent probes or utilized in photothermal imaging, facilitating magneto-optical multimodal imaging that operates without the need for continuous excitation or interference of autofluorescence (Key and Leary, 2014). These advances offer more comprehensive imaging information, further reinforcing the potential of MNMs in precision medical diagnostics and image-guided therapy.

Magnetic nanomaterials, owing to their controllable magnetic hyperthermia effect, targeted delivery capabilities, and multimodal therapeutic properties, have demonstrated significant potential in personalized therapy and theranostics (Accogli et al., 2021). Their therapeutic mechanisms primarily include magnetic hyperthermia therapy (MHT), photothermal therapy (PTT), magnetic-targeted drug delivery (MTDD), and magnetically mediated gene or immunotherapy. By integrating real-time medical imaging, these approaches enable precise interventions and individualized treatment adjustments. Among them, MHT is the most widely used approach, in which magnetic nanomaterials generate localized heat under an alternating magnetic field, inducing thermal damage or apoptosis in tumor cells within the temperature range of 42°C–45°C, thus improving therapeutic efficacy (Salloum et al., 2008). Furthermore, PTT employs magnetophotothermal composite nanomaterials to convert near-infrared (NIR) light into heat energy, facilitating tumor ablation (Liang et al., 2021). When combined with MHT, this approach can further improve treatment efficiency through synergistic effects (Du et al., 2021). In the context of personalized therapy, the therapeutic intensity, mode of action, and delivery pathway of magnetic nanomaterials can be optimized according to the pathological characteristics of the patient. For instance, the localized heat generated by magnetic nanomaterials under a magnetic field can be precisely modulated to regulate the tumor microenvironment. Depending on tumor type, location, and patient tolerance, the magnetic hyperthermia dose can be adjusted for precise thermal ablation. Meanwhile, MTDD technology uses magnetic field guidance to facilitate the targeted delivery of drug-loaded magnetic nanomaterials to tumor sites, thus improving therapeutic efficacy while minimizing systemic side effects (Mok and Zhang, 2013).

Despite significant advancements in applying magnetic nanomaterials for imaging and therapy, their clinical translation remains challenging. For instance, magnetic nanomaterials tend to aggregate or undergo oxidation in biological fluids, leading to magnetic signal attenuation, compromising MRI contrast enhancement and the efficacy of magnetic hyperthermia (MHT), weakening the synergy between imaging and therapy (Figueiredo et al., 2022). Furthermore, precise control of thermal dosage in MHT is difficult, as uneven distribution of nanomaterials may cause localized overheating or inadequate treatment, while high concentrations of nanomaterials can induce T2 signal attenuation, reducing the quality of the MRI image (Shu et al., 2021). In targeted delivery, the limited penetration depth of external magnetic fields hinders the effective guidance of nanomaterials to deep-seated lesions, and their rapid clearance by the reticuloendothelial system (RES) further reduces therapeutic efficiency (Xu S. et al., 2023). Therefore, enhancing magnetic field control precision and developing smart, responsive magnetic nanomaterials are critical for advancing their clinical application in imaging and therapy.

This review provides a comprehensive and systematic overview of recent advancements in the integration of medical imaging and clinical therapy utilizing MNMs. Initially, various types of MNMs, including iron oxide nanoparticles, magnetic metal nanomaterials, composite MNMs and others, are introduced, with a focus on their functional properties and advantages in medical imaging and therapeutic applications (Section 2). Subsequently, the role of MNMs as novel contrast agents in radiological imaging modalities such as MRI and CT is examined, along with their applications in precision oncology. Key therapeutic strategies involving MNMs, including magnetic targeted drug delivery and magnetic hyperthermia, are discussed, as well as their synergistic effects with conventional treatment modalities, such as the enhanced therapeutic efficacy achieved through the combination of magnetic hyperthermia with chemotherapy and radiotherapy (Sections 3, 4). Furthermore, the review explores imaging-guided therapeutic strategies based on MNMs (Section 5). By gaining insights into the latest advances in the application of magnetic nanomaterials in biomedical imaging and cancer therapy, we aim to provide new directions for the optimization and clinical translation of nanomaterials.

## 2 Magnetic nanomaterials

MNMs are usually magnetized composites made of metals and their oxides, such as iron, nickel, cobalt, etc. These are aligned on the nanoscale to form magnetic domains, which produce magnetic properties. The properties of MNMs derive to a large extent from their physicochemical properties, average size, and morphology. For example, size affects the strength of the magnetic properties of the particles. MNMs exhibit superparamagnetism as their size decreases to close to the diameter of a single magnetic domain, which means that the magnetization strength decreases to zero when the applied magnetic field is removed (Zhu et al., 2018; Jun et al., 2005), due to significant changes in the thermal motion and magnetization behavior of particles at the nanoscale. This ability to interact with external magnetic fields allows them to be remotely and precisely modulated, thus opening up a wide range of possibilities for the development of biomedical technologies aimed at improving the understanding, diagnosis, and treatment of different diseases (Dobson, 2008). Table 1 illustrates the structures of some of the most recently designed MNMs and their synergistic imaging and therapeutic capabilities, which may help to better understand the effect of composition on the performance of MNMs and to design the structures of MNMs on demand in the future in order to maximize their performance and improve diagnostic and therapeutic efficiency. Depending on the magnetic properties and composition, there are several main types of MNMs.

**TABLE 1:** Basic information on disease imaging and therapeutic research based on magnetic nanomaterials.

Reference	Year	Magnetic material	Imaging modality	Disease
Pan et al. (2016)	2016	UCNPs–yolk–shell NPs	UCL/MRI (T2WI)	Breast Cancer
Huang et al. (2016)	2016	Tween-SPIONs	MRI (T2WI)	Brain Tumors
Zhang et al. (2016)	2016	CTX-NC	MRI (T2WI)	Early Glioma
Liu et al. (2016)	2016	Fe0.6 Mn0.4 O	MRI (T1WI/T2WI)	MCF-7 Breast Cancer, Orthotopic Glioma
Li et al. (2017)	2017	Magnetic graphitic NPs	MRI (T2WI)	*Helicobacter Pylori* Infection
Kale et al. (2017)	2017	${\text{Fe}}_{3}{\text{O}}_{4}$ @GdPB	MRI (T2WI)	Neuroblastoma
Liu et al. (2018)	2018	${\text{Ta}}_{4}{\text{C}}_{3}$ -IONP-SPs	CT/MRI (T2WI)	Breast Cancer
Abedin et al. (2018)	2018	PLL-Au- ${\text{Fe}}_{3}{\text{O}}_{4}$ NPs	MRI (T2WI)	Breast Cancer
Lee et al. (2018)	2018	NF-SIONs	NIR/MRI (T2WI)	Glioblastoma
Jermy et al. (2019)	2019	10 wt%SPIONs/S-16-A-Cp	MRI (T2WI)	Colon and Cervical Cancer
Shi et al. (2019)	2019	Gd-doped CuS NPs (T-MAN)	NIR/MRI (T2WI)	Gastric Cancer
Zhong et al. (2019)	2019	α - ${\text{Fe}}_{2}{\text{O}}_{3}$ @Au	MRI (T2WI)	Breast Cancer
Chen et al. (2020)	2020	CuS- ${\text{NiS}}_{2}$	MRI (T2WI)	Gastric Carcinoma
Das et al. (2020)	2020	Zn-SPIONs	MRI (T2WI)	Glioblastoma
Xu et al. (2020a)	2020	Au- ${\text{Fe}}_{3}{\text{O}}_{4}$ @PDA-PEG-DTPA-Gd	CT/MRI (T1WI/T2WI)	Triple-negative Breast Cancer
Shen et al. (2020)	2020	ES-GON-rBSA-LF- ${\text{RGD}}_{2}$	MRI (T2WI)	Glioblastoma
Kermanian et al. (2021)	2021	Fe-Si-In	MRI (T2WI)	Acute Liver Failure
Yang et al. (2021)	2021	PFH@PLGA/ ${MnFe}_{2}{\text{O}}_{4}$ -Ram	MRI (T2WI)/PAI/US	Atherosclerosis
Saraswathy et al. (2021)	2021	P-SPIONs	NIR/MRI (T2WI)	Liver Fibrosis
Luque-Michel et al. (2021)	2021	SPION-DOX-PNP	MRI (T2WI)	Glioma
Wang et al. (2022a)	2022	IMQ@IONs/ICG	MRI (T2WI)	Pancreatic Cancer
Liu et al. (2022)	2022	FePPy- ${\text{NH}}_{2}$ NPs	MRI (T2WI)/PAI	Bladder Cancer
Park et al. (2022)	2022	KDR-MN	MRI (T2WI)	Endometriosis
Zhou et al. (2023)	2023	MHD	MRI (T2WI)	Breast Cancer
Hasani et al. (2023)	2023	${\text{Fe}}_{3}{\text{O}}_{4}$ - β CD-Pep42-DOX	MRI (T2WI)	Breast Cancer
Nazeer et al. (2023)	2023	ASPION-AT	NIR/MRI (T2WI)	Liver Fibrosis, Hepatocellular Carcinoma
Dhawan et al. (2023)	2023	FeAu@MOF	MRI (T2WI)	Oral Squamous Cell Carcinoma
Tao et al. (2024)	2024	MFe3O4-labeled EGFP-NPCs	MRI (T2WI)	Glioma
Gao et al. (2024)	2024	α - ${\text{NaErF}}_{4}$ @NaY $F_{4}$ NPs	NIR/CT/US	Breast Cancer
Zhang et al. (2024b)	2024	SPFeNOC	NIR/MRI (T2WI)	Bone Metastasis

### 2.1 Superparamagnetic iron oxide nanoparticles (SPIONs)

Superparamagnetic Iron Oxide Nanoparticles (SPIONs) consist of magnetic hematite (such as γ - ${\text{Fe}}_{2}{\text{O}}_{3}$ ), magnetite ( ${\text{Fe}}_{3}{\text{O}}_{4}$ ), and other metallic ferrites (Bárcena et al., 2009). As one of the most widely used MNMs in current clinical applications, it has become the core carrier of the integrated multimodal diagnostic and therapeutic platform under its unique superparamagnetism and excellent biocompatibility. The crystal size of SPIONs is usually controlled to be less than 20 nm in size, to ensure that there is no residual magnetism after the external magnetic field is withdrawn, thus avoiding the risk of *in vivo* aggregation. It can improve the biocompatibility and tumor targeting of nanoparticles through surface modification (such as PEGylation, antibody modification, etc.), achieve tumor-specific enrichment, and significantly reduce the uptake of non-target organs (Mok and Zhang, 2013; Xiong et al., 2012).

In diagnostic imaging, SPIONs have been widely studied and applied as MRI contrast agents, demonstrating versatility in both T1 and T2-weighted imaging. Traditionally, SPIONs have been predominantly used as T2 contrast agents, where their strong magnetic moments shorten the transverse relaxation time (T2), resulting in significant signal attenuation in T2-weighted imaging and thus improving contrast (Lin et al., 2015). This property allows for the high-sensitivity detection of small tumor lesions. In addition, ultrasmall SPIONs (USPIOs) and surface-engineered SPIONs can also serve as T1 contrast agents, reducing longitudinal relaxation time (T1) and generating positive contrast in T1-weighted imaging. This dual mode imaging capability provides complementary diagnostic information, improving the accuracy of lesion identification. Furthermore, the functionalizable surface of SPIONs enables multimodal imaging by integrating MRI with optical imaging, PET, or CT through surface conjugation with fluorescent dyes or radionuclides (e.g., ^64^Cu). This multimodal coupling offers comprehensive diagnostic insights into complex lesions, allowing precise localization and characterization of pathological features (Yang et al., 2017). In terms of drug delivery, as inorganic nano drug carriers approved for clinical use, SPION has shown multimodal synergistic potential in the integrated diagnosis and treatment of tumors due to its advantages such as superparamagnetism, low toxicity, metabolic control, and surface functionalization. The magnetic response properties of SPIONs provide a precise means of spatial control for targeted therapy. Guided by an external magnetic field, drug-loaded SPIONs can achieve local drug enrichment at the tumor site, thus breaking the systemic toxicity bottleneck of conventional chemotherapy (Senapati et al., 2018). In the field of tumor thermotherapy, SPIONs provide a new way for local thermal ablation therapy by converting alternating magnetic field (AMF) energy into thermal energy through the hysteresis loss effect (Klapproth et al., 2020).

### 2.2 Magnetic metal nanomaterials

MNMs composed of single metallic elements, such as iron, cobalt, and nickel, have attracted attention due to their high saturation magnetization, superparamagnetic behavior at the nanoscale, and tunable magnetic properties. However, their application in biomedicine is often limited by their intrinsic chemical instability. These transition metals exhibit strong reactivity and are prone to oxidation in aqueous and oxygen-rich environments, leading to the formation of oxide layers that can alter their magnetic properties, reduce biocompatibility, and limit long-term stability. To mitigate these challenges, protective coatings such as silica, gold, carbon, or polymer layers are commonly employed to form core-shell structures, which enhance their chemical stability, dispersibility, and biocompatibility (Peng et al., 2006). Despite their susceptibility to oxidation, monometallic MNMs offer several advantages over their oxide counterparts. Their higher intrinsic magnetization enables stronger magnetic responses, which is particularly beneficial in magnetic hyperthermia, targeted drug delivery, and contrast-enhanced MRI applications (Peng et al., 2006; Shao and Ren, 2020). Furthermore, their surface properties, including size-dependent catalytic activity and enhanced electron transfer capabilities, make them valuable in biosensing, nanozymes, and environmental remediation (Wong et al., 2021).

### 2.3 Magnetic alloy nanomaterials

Magnetic Alloy Nanomaterials are synthesized by combining two or more different pure metal elements into the particle core. Compared to single magnetic alloy nanomaterials that only acquire the properties of their constituent metals, metal alloys and bimetallic magnetic alloy nanomaterials have attracted widespread attention due to their ability to exploit the synergistic functional effects of each metal (Thangudu et al., 2023). When two or more metal atoms are combined to form an alloy, chemical interactions occur between them, which not only enhances the stability of the overall structure and improves resistance to external chemical degradation sources, but also can reduce the toxicity of the particles by adjusting the ratio of the metal components (Sun et al., 2008; Thangudu et al., 2023). In addition, the introduction of a second or third metal changes the magnetic distribution of the atoms, thereby enhancing the superparamagnetic properties of MNMs without increasing the particle size (Pardo et al., 2020; Sun et al., 2008). In the medical field, the greatest advantage of alloy MNMs is their significant improvement in magnetic properties. This improvement not only enhances the effect of MRI but also opens up the possibility of multimodal imaging. The alloy structure also provides MNMs with additional protection against chemical degradation in the body (such as oxidation), further extending their stability and application time *in vivo*. Metal alloys and bimetallic nanomaterials often exhibit enhanced magnetic, catalytic, optical, and electronic properties compared to single metal nanomaterials. This synergistic effect enables alloy nanomaterials to exhibit superior performance in biomedical applications, especially in imaging and therapy.

For example, combining iron (Fe) with tin (Sn) with good biocompatibility to form alloy nanomaterials can significantly reduce the inherent toxicity of iron, without significantly affecting liver and kidney function, while maintaining its performance as a MRI contrast agent, making it a potential candidate for clinical applications of MRI contrast agents (Thangudu et al., 2023). In addition, iron-gold (Fe-Au) alloy nanoparticles have attracted extensive attention in medical imaging, magnetic separation, and nanodiagnosis and treatment due to their excellent magnetic properties and biocompatibility. In medical imaging, since iron-gold alloy nanoparticles (Fe-Au) exhibit lower magnetization than iron oxide nanoparticles of similar size, their potential transverse relaxation to longitudinal relaxation rate ratio (r2/r1) is lower, which is conducive to its use as a contrast agent T1 for MRI, thus improving the efficiency of imaging diagnosis (Liang et al., 2024). By combining Ti@FeAu nanoparticles with Angiopep-2 (a peptide that can penetrate the blood-brain barrier and target glioma cells), Ti@FeAu-Ang nanoparticles showed specific targeting of glioma cells and higher cellular uptake rate, exhibiting significant tumor inhibitory effects without significant toxicity to major organs, indicating its potential in cancer diagnosis and treatment (Thirumurugan et al., 2022).

### 2.4 Magnetic rare earth metal nanoparticles

Rare earth metals (such as lanthanum, neodymium, europium, etc.) and their oxides or alloys can synthesize nanomaterials with strong magnetism under certain conditions. These rare earth nanoparticles (RENPs) exhibit high chemical stability, strong resistance to photobleaching, and good biocompatibility. In particular, through the clever core-shell structure design, they can integrate multimodal imaging and treatment functions (Lin et al., 2025). In optical imaging, the advantages of RENPs are particularly significant, including high resolution, low toxicity, biocompatibility, and specific targeting capabilities achieved through surface functionalization. These characteristics make them have great application potential in non-invasive imaging in cancer immunotherapy (Gao et al., 2024). In addition, RENPs increase the local radiation dose at the tumor site with their high magnetic moment, long electron relaxation time, and high X-ray absorption coefficient, providing high-contrast imaging effects in MRI, and can achieve multimodal imaging of MRI, optical imaging, and CT imaging (Wei et al., 2022). For example, gadolinium ( ${\text{Gd}}^{3+}$ ) doped nanoparticles, Gadolinium (e.g., ${\text{NaGdF}}_{4}$ ) are used not only for optical imaging, but also as MRI contrast agents, while multimodal nanoprobe ${\text{CaF}}_{2}$ : Y, Gd, Nd NPs combine NIR-II imaging and MRI to achieve high contrast detection of tumors (Skripka et al., 2017; Yu et al., 2022). RENPs can also be used to monitor neuronal activity in real time, especially through upconversion nanoparticles to achieve long-term neuronal activity tracking (Wei et al., 2022). In addition, neodymium-doped nanoparticles, with their unique near-infrared luminescence properties, can achieve real-time monitoring of subcutaneous temperature and serve as efficient photothermal agents for heating tumor tissues. This dual function strongly demonstrates the significant advantages of RENPs in the combined application of imaging and therapy (Rocha et al., 2016).

### 2.5 Magnetic carbon nanomaterials

Magnetic carbon-based nanoparticles (MCNPs) have shown broad application prospects in the biomedical field due to their high specific surface area, good chemical and thermal stability, and unique magnetic properties. The core components of this type of material include carbon nanotubes, graphene and its various derivatives, such as graphene oxide (GO), reduced graphene oxide (rGO), and graphene quantum dots (GQD). The honeycomb lattice structure formed by hybridization gives these materials extremely high carrier mobility and huge theoretical specific surface area (Itoo et al., 2022). On this basis, by cleverly incorporating magnetic elements such as iron, cobalt, and nickel, MCNPs not only completely retain the original excellent biocompatibility and chemical stability of carbon-based materials, but also have the high magnetic saturation and superparamagnetism of MNMs (Gostaviceanu et al., 2024). These properties enable it to exhibit extraordinary performance in many aspects such as multimodal imaging, cell labeling, and targeted drug delivery. In terms of imaging, MCNPs cleverly combine the strong absorption properties of carbon-based materials in the near-infrared (NIR-II window) region with the contrast enhancement ability of magnetic components in MRI (T2 relaxation rate can exceed 200 ${\text{mM}}^{-1}{\text{s}}^{-1}$ ), providing a highly sensitive tool for early diagnosis of tumors (Reimer and Ferucarbotran, 2003). At the same time, the magnetic component achieves precise targeting of drugs under the guidance of an external magnetic field. In the field of drug delivery, graphene’s π electron system and rich surface reaction sites provide extremely high capacity for drug loading (drug loading rate can exceed 91%) (Guo and Mei, 2014; Shirvalilou et al., 2018; Rodrigues et al., 2018). At the same time, they also have excellent high thermal conductivity, which promotes their light absorption in the near-infrared window, which lays the foundation for their application in photothermal therapy (Wang Y. et al., 2022).

### 2.6 Magnetic nanocomposites

Magnetic nanocomposites are multi-component materials that combine the advantages of multiple materials (such as metals, oxides, polymers, etc.), and they have shown powerful functions and application potential in many fields. The advantages of nanomagnetic composites are mainly reflected in multifunctionality, performance optimization, and biocompatibility. First, multi-functionality is one of its major features. Composite materials can simultaneously have imaging, treatment, and targeting functions, providing the possibility of accurate diagnosis and treatment of diseases. For example, by combining iron oxide with materials such as gold and silver, the prepared composite particles not only significantly enhance the magnetism, but also greatly improve the drug delivery ability (Abedin et al., 2018). Secondly, by combining different components, the magnetism, stability, and biocompatibility of the composite material can be optimized, making it more suitable for various complex biological environments. For example, by modifying the surface with aptamers or antibodies, the composite material can achieve specific targeting, further improving the accuracy and effect of treatment (Hasani et al., 2023). At the same time, the application of modified ingredients such as polyethylene glycol (PEG) not only improves the dispersibility and stability of the particles, but also reduces the clearance of macrophages, thereby enhancing its accumulation and therapeutic effect at the tumor site (Xu Q. et al., 2020).

In terms of application, magnetic nanocomposites have demonstrated excellent performance in many fields such as MRI imaging, drug delivery, and magnetic hyperthermia therapy. As contrast agents for MRI imaging, these composites can provide high-resolution images to assist doctors in making accurate diagnoses (Luo et al., 2022). At the same time, during the drug delivery process, they can carry and accurately deliver drugs to the lesion site, significantly improving the treatment effect (Taheri-Ledari et al., 2023). In addition, in magnetic hyperthermia therapy, the composites can generate local heat under an alternating magnetic field, effectively killing cancer cells, showing good therapeutic potential (Illés et al., 2020).

In summary, MNMs include many different types of materials, each of which shows adaptability to specific medical applications based on its unique magnetic characteristics, fine structure, and functionalization capabilities. The diversity of MNMs makes them have broad prospects in precision medicine. In the field of biomedical imaging, these materials can significantly improve the resolution and accuracy of imaging; in terms of treatment, they show great potential as targeted drug carriers or directly used in therapies such as magnetic hyperthermia. More importantly, the diversity and adjustability of MNMs provide a new perspective and strategy for the integration and innovation of multimodal imaging and treatment technologies, and promote medical diagnosis and treatment methods to move towards a more accurate and efficient direction.

### 2.7 Promising materials beyond iron oxide-based nanoparticles

While superparamagnetic iron oxide nanoparticles (SPIONs) have been the most extensively studied and clinically translated magnetic nanomaterials, several alternative material classes have recently emerged, offering complementary advantages in imaging, therapy, and multifunctional platform development.

Magnetic carbon-based nanostructures, including graphene oxide (GO), carbon nanotubes (CNTs), and carbon dots decorated with magnetic domains (e.g., Fe, Co, Ni), represent a promising class of MNMs. These hybrids combine excellent photothermal conversion efficiency, large surface areas for drug loading, and strong near-infrared (NIR) absorbance, which are advantageous for multimodal imaging and synergistic therapies such as photoacoustic imaging (PAI) and photothermal therapy (PTT) (Ji et al., 2021). Moreover, their -conjugated frameworks facilitate electron transport, enabling electrochemical biosensing and responsive drug release strategies. Surface functionalization of magnetic carbon nanomaterials allows precise targeting and improved biocompatibility. However, concerns regarding their long-term biodegradability and potential cytotoxicity need to be addressed before clinical translation.

Organic–inorganic hybrid nanomaterials, such as magnetic metal–organic frameworks (MOFs) and conjugated polymer–iron oxide hybrids, provide versatile platforms combining tunable porosity, high drug-loading capacity, magnetic responsiveness, and biodegradability. For instance, magnetic MOFs can serve as reservoirs for drugs and photosensitizers, while their magnetic cores enable MRI guidance. These systems can be engineered to respond to multiple stimuli (e.g., pH, enzymes, light) for controlled drug release and targeted therapy (Lu et al., 2018). Nevertheless, maintaining magnetic stability while ensuring biodegradability and non-toxicity remains a key challenge for such hybrid structures.

Magnetic alloys (e.g., FeCo, FePt) and rare-earth element-doped nanoparticles (e.g., Gd-doped ferrites, Dy-doped oxides) exhibit enhanced magnetic properties compared to conventional iron oxides. FeCo nanoparticles, for instance, possess higher saturation magnetization, which improves magnetic hyperthermia efficiency and magnetic targeting capabilities (Liu et al., 2021). Gd-doped systems offer both MRI T1 and T2 contrast enhancement, providing dual-mode imaging opportunities (Cao et al., 2017). However, these materials must be carefully designed to minimize potential toxicity associated with metal ion leaching and to ensure colloidal stability under physiological conditions.

Collectively, these emerging material systems significantly broaden the design space for magnetic nanomaterials, enabling customized theranostic agents tailored to specific clinical needs, imaging modalities, and therapeutic strategies. Future research should focus on optimizing the balance between magnetic performance, biocompatibility, biodegradability, and functional versatility to accelerate their translation into clinical practice.

## 3 Application of magnetic nanomaterials in multimodal imaging

**FIGURE 1:**
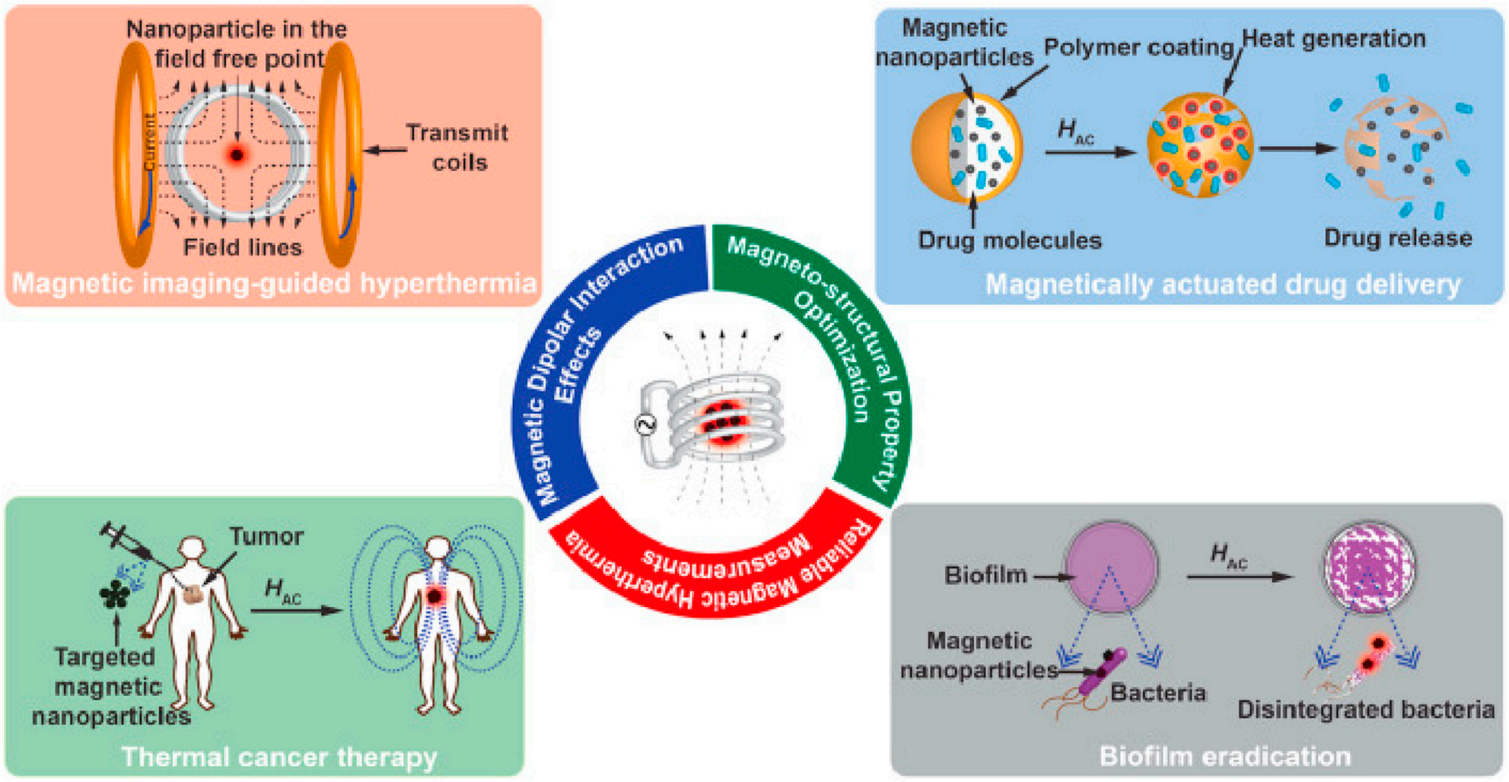
Schematic illustration of magnetic nanoparticle-mediated hyperthermia for cancer therapy (Abenojar et al., 2016).

Medical imaging plays a vital role in early diagnosis of diseases, condition assessment and therapeutic efficacy monitoring. In this section, we focus on the application of MNMs in different imaging techniques, highlighting their potential in multimodal imaging (Figure 1).

### 3.1 Application in MRI

Magnetic resonance imaging (MRI), as a non-invasive imaging technique with high soft tissue resolution and no ionizing radiation, has unique advantages in tumor diagnosis (Seo et al., 2022). However, conventional MRI has insufficient contrast in some cases, and the use of contrast agents is often required to improve image quality and diagnostic accuracy (Wang et al., 2024).

MMNMs exhibit magnetic responsiveness, allowing them to generate strong magnetic signals that significantly enhance contrast in MRI (Wang et al., 2024). Among them, ferrite nanoparticles, particularly ultrasmall superparamagnetic iron oxide nanoparticles (USPIOs), are widely utilized as T2 contrast agents. By shortening the T2 relaxation time, these nanoparticles produce negative contrast signals, making them highly effective for imaging organs rich in the reticuloendothelial system (RES), such as the liver, spleen, and lymph nodes, as well as for diagnosing tumors and inflammatory conditions (Xie Q. et al., 2024). However, T2 contrast agents can be affected by magnetic susceptibility artifacts and signal ambiguity, which has led to increasing research on T1 contrast agents. To enhance T1 relaxation effects and generate positive contrast signals, researchers have developed strategies involving precise control of particle size, crystal phase engineering, and surface functionalization. These advancements have improved the clinical utility of ferrite-based MRI contrast agents, enabling higher-resolution imaging and more accurate disease detection (Ni et al., 2016; Zeng et al., 2018).

**FIGURE 2:**
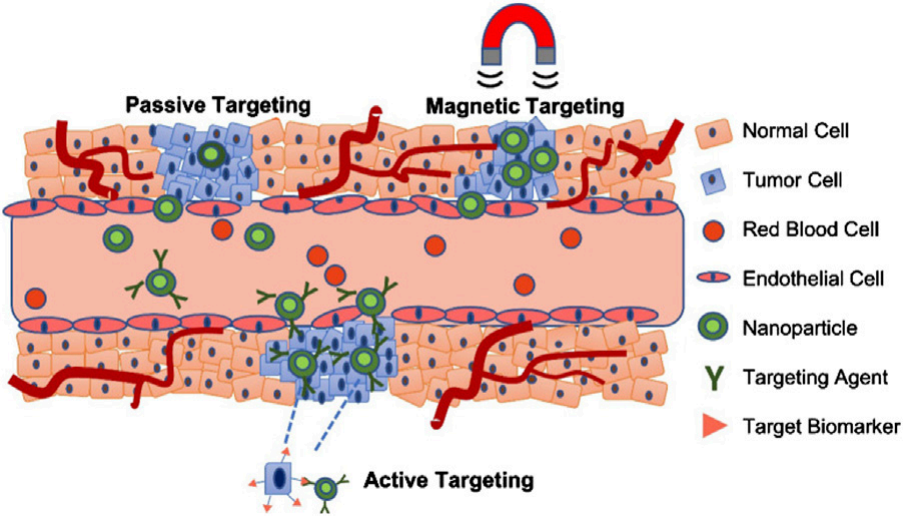
Passive, active and magnetic targeting strategies utilized to enhance the accumulation and efficacy of MRI-traceable, theranostic nanoparticles for targeted cancer treatment. Passive targeting exploits the leaky vasculature and poor lymphatic drainage in the tumor, while active targeting also exploits specific interactions between a targeting agent (e.g., antibody, peptide or aptamer) on the nanoparticle and a nearby biomarker on the target cancer cell. Magnetic targeting, on the other hand, utilizes an externally applied magnetic field to retain magnetic nanomaterials at the tumor site (Anani et al., 2021).

In addition to enhancing contrast, surface modification techniques further improve the targeted imaging capabilities of MNMs. By conjugating nanoparticles with functional molecules such as PEG, antibodies, or peptides, they can selectively bind to specific tissues or cells, thereby increasing image resolution and diagnostic accuracy. For instance, antibody-functionalized iron oxide nanoparticles have been successfully applied in the imaging of various cancers, including glioma, breast cancer, and pancreatic cancer (Tao et al., 2024; Zhou et al., 2023; Zhu et al., 2022). This targeted imaging strategy not only facilitates early lesion detection but also provides critical insights for personalized treatment planning. To achieve efficient tumor localization, different nanoparticle targeting strategies have been developed, including passive targeting, active targeting, and magnetic targeting, each employing distinct mechanisms to enhance the accumulation of imaging and therapeutic agents at the diseased site, as shown in Figure 2.

### 3.2 Application in CT imaging

MNMs show unique advantages and promising applications as contrast agents in CT imaging. MNMs, especially ferrite (e.g., ${\text{Fe}}_{3}{\text{O}}_{4}$ , ${\text{Fe}}_{3}{\text{O}}_{4}$ @Au, etc.) and superparamagnetic materials (e.g., superparamagnetic iron oxide nanoparticles, SPIONs), have become indispensable contrast agents in CT imaging due to their high density and good X-ray attenuation properties. Compared to traditional iodine-based contrast agents, they provide efficient contrast enhancement while avoiding side effects such as allergic reactions or kidney damage that may be triggered by iodine-based contrast agents (Chen et al., 2021). Through surface modification techniques, such as antibody modification and PEG modification, MNMs can achieve targeted enhancement and enrichment in specific lesion areas, thus improving image quality and localization accuracy. In addition, some MNMs, such as ferrite particles, have lower biotoxicity compared to iodine-based contrast agents and can circulate in the body for a long period, providing more imaging opportunities for patients, which is crucial for continuous monitoring of the disease and evaluation of treatment (Chen et al., 2021). In practical applications, MNMs can help doctors accurately assess the structure and pathology of blood vessels, which is of great significance for the diagnosis of diseases such as atherosclerosis or thrombosis (Chen et al., 2021).

### 3.3 Application in PET and SPECT imaging

MNMs in combination with positron emission tomography/single photon emission computed tomography (PET/SPECT) have demonstrated unique advantages in the field of medical imaging. PET/SPECT characterizes the biological functions of the body at the molecular level, allowing for detailed understanding of disease and individualized treatment of patients (De Jong et al., 2009). MNMs can significantly enhance the sensitivity of PET or SPECT imaging in combination with radiolabeling. Specifically, by combining MNMs, with PET tracers, researchers were able to create a novel tracer for dual-modality imaging (Bashir et al., 2015; Swierczewska et al., 2011). This tracer not only inherits the high sensitivity and whole-body imaging capabilities of PET imaging for precise quantification and localization of biological processes such as cancer cells, but also introduces magnetic properties that make these nanomaterials suitable for both magnetic resonance imaging (MRI), thus combining the high spatial resolution of MRI (Yang et al., 2017).

In addition, during PET imaging, short-lived radionuclides are labeled on MNMs that travel with the bloodstream to various parts of the body. By measuring the distribution and metabolism of these labeled substances in the body, information on the structure and function of the organism can be obtained non-invasively. For example, Chen et al. synthesized ^64^Cu-labeled nanomaterials of different sizes and successfully investigated the effect of nanoparticle size on the localization of lymph nodes, showing the practicality of this labeling method (Yang et al., 2017).

### 3.4 Application in optical imaging

Fluorescence imaging involves the excitation of certain fluorophores by an external light source and the detection of the emitted light using a highly sensitive charge-coupled device camera. Fluorophores can be endogenous molecules (e.g., hemoglobin) or exogenous molecules (e.g., synthetic optical probes) (Key and Leary, 2014). Compared with other existing imaging techniques, optical imaging, especially near-infrared fluorescence imaging, which has the advantages of low cost, fast feedback, high sensitivity, and no radiation hazard, has attracted considerable attention in recent years (Rocha et al., 2016).

Integrating MNMs with techniques such as near-infrared fluorescence imaging can significantly improve imaging performance. MNMs can be modulated by an external magnetic field to achieve precise localization, while near-infrared spectroscopic imaging uses excitation light above 700 nm to break through the optical barrier of biological tissues, enabling penetration depths of 1–2 cm and reducing scattering interference (Du et al., 2024). This dual-modality synergy effectively compensates for the shortcomings of traditional imaging techniques. For example, MRI has the ability of deep imaging but lacks temporal resolution, while single fluorescence imaging is rapid but limited by the penetration depth (Key and Leary, 2014). In addition to conventional imaging modalities such as MRI and CT, the development of novel nanomaterials with emission beyond 1,000 nm, such as emissive ruthenium (II) metallacycles, has opened new possibilities for high-resolution, deep-tissue imaging in the NIR-II window (Xu et al., 2022).

### 3.5 Multimodal imaging design challenges and optimization strategies

While MNMs have shown great promise across various imaging modalities, the integration of MRI, CT, PET, and optical imaging functionalities within a single platform presents substantial design challenges, as different imaging techniques impose distinct and sometimes conflicting requirements on material properties.

For MRI, particularly T2-weighted imaging, high magnetic susceptibility and stable dispersion are essential to maximize signal contrast; yet, excessive nanoparticle concentrations may induce T2 signal quenching, resulting in image darkening and reduced diagnostic clarity. In contrast, CT imaging demands the incorporation of high atomic number (Z) elements such as gold or bismuth to enhance X-ray attenuation. The addition of heavy elements inevitably increases particle density and size, which can adversely affect magnetic performance, circulation time, and biocompatibility. PET imaging further requires stable and efficient radiolabeling, introducing chemical modifications that may alter nanoparticle surface chemistry and impact magnetic responsiveness. Optical imaging, such as fluorescence imaging, photoacoustic imaging (PAI), and near-infrared (NIR) imaging, imposes additional design considerations. Efficient optical imaging relies on strong light absorption, high quantum yield, and minimal background interference. However, the dense metallic or magnetic cores essential for MRI or CT can introduce optical quenching effects through non-radiative energy transfer or scattering, thereby diminishing fluorescence signals. Moreover, the requirement for optical transparency at specific wavelengths (e.g., NIR-I, NIR-II windows) may conflict with the material’s magnetic or X-ray attenuation properties.

To overcome these multifaceted challenges, several advanced design strategies have been proposed. Core–shell architectures, where the magnetic core is coated with an optically active or high-Z element-containing shell, can spatially decouple magnetic, radiodense, and optical functionalities. Hybrid nanoparticle systems, such as iron oxide–gold composites or iron oxide–fluorophore conjugates, allow independent tuning of each imaging modality while maintaining overall biocompatibility. Stimuli-responsive MNMs capable of modulating their optical or magnetic properties in response to tumor microenvironmental cues (e.g., pH, redox gradients, enzymatic activity) offer dynamic imaging enhancement without systemic interference. Additionally, surface engineering strategies such as PEGylation, zwitterionic modification, or the integration of optical spacers can reduce optical quenching and improve nanoparticle pharmacokinetics.

Looking forward, the rational integration of multimodal functionalities should prioritize the development of smart, adaptive nanoplatforms capable of selectively activating specific imaging modes based on clinical demands. Achieving such intelligent control will be pivotal for unlocking the full potential of MNMs in precision diagnostics, image-guided therapy, and personalized medicine.

## 4 Magnetic nanomaterials in therapy

Over the past few decades, MNMs have become important tools in the biomedical field due to their unique superparamagnetism, tunability (their electrical, optical, and magnetic properties can be adjusted as needed), stability, biocompatibility, and large surface area that can be easily functionalized. They have been widely used in biomedical applications including MHT, PTT, MTDD, etc., showing their unlimited potential for biomedical applications.

### 4.1 Magnetic hyperthermia (MHT)

**FIGURE 3:**
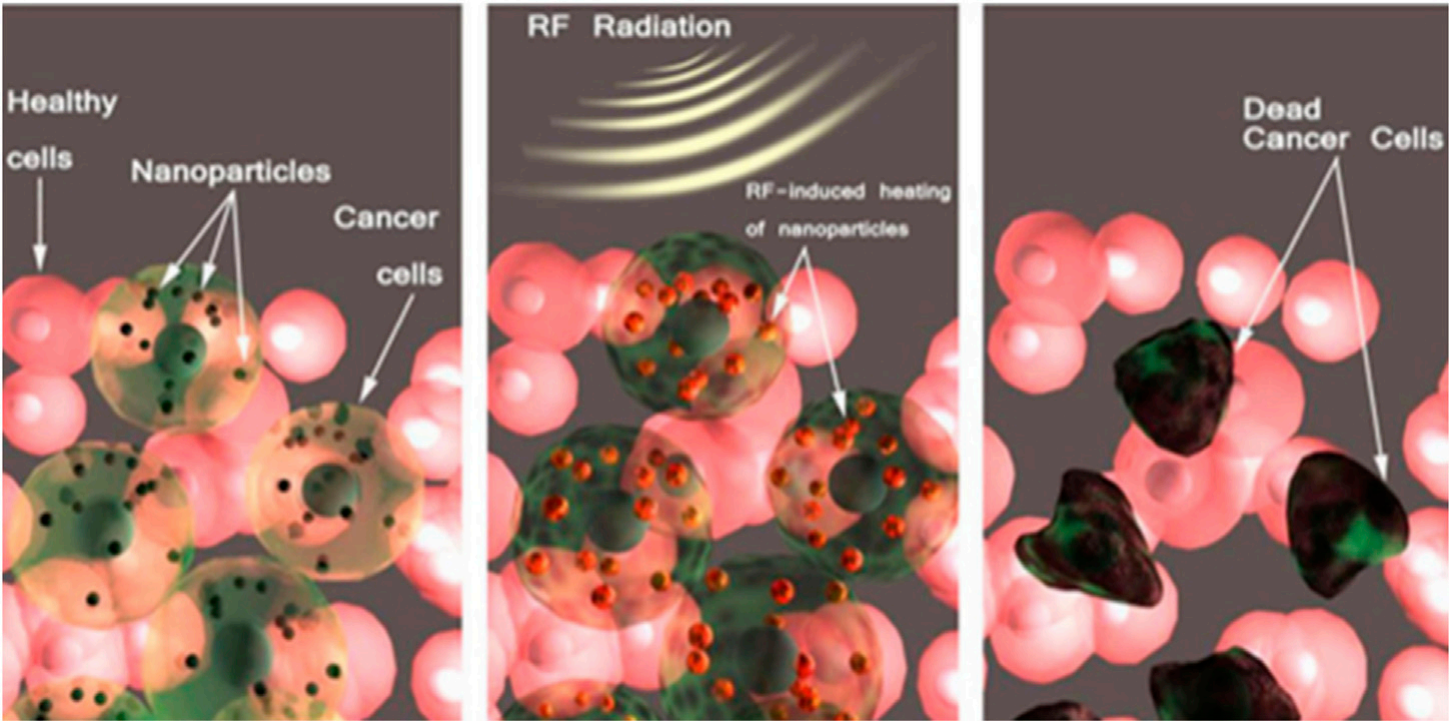
Schematic representation of the magnetic hyperthermia treatment procedure (Fatima et al., 2021).

MNMs generate heat when exposed to an alternating magnetic field (AMF), a phenomenon widely utilized in tumor therapy known as MHT (Rosensweig, 2002). MNMs can be administered either intravenously or directly into the tumor site, where they preferentially accumulate (Salloum et al., 2008). Upon application of the AMF, the magnetic moments of the MNMs continuously realign with the oscillating field, leading to hysteresis loss and the conversion of magnetic energy into heat (Figure 3). This localized heating effect raises the temperature within the tumor to a therapeutic range, inducing irreversible cellular damage while sparing surrounding healthy tissues (Zhang et al., 2023). Due to the heightened thermal sensitivity of cancer cells, exposure to temperatures between 42°C and 45°C triggers apoptosis or necrosis, effectively impairing tumor growth (Salloum et al., 2008). This selective heating capability makes MHT a minimally invasive and highly efficient approach for tumor treatment.

The efficiency of this heat generation is determined by the specific absorption rate (SAR), which is closely related to the saturation magnetization strength (Ms) of the nanoparticles (Deatsch and Evans, 2014). It has been shown that the therapeutic effect of MHT can be significantly optimized by adjusting the size, shape, and surface modification of the nanoparticles as well as the frequency and intensity of the alternating magnetic field (Tang et al., 2021). Superparamagnetic iron oxide nanoparticles (SPIONs) are commonly used MHT agents, but their low Ms (60 emu/g) results in limited SAR values (Lu et al., 2007; Kharisov et al., 2012; Cho et al., 2006). To improve the SAR value, researchers have developed magnetic materials with higher Ms, such as pure iron nanoparticles (FeNPs, Ms of 218 emu/g), and improved their stability and biocompatibility by surface modification (e.g., coating with gold or iron oxide shell layers) (17–19). Increasing the size of nanoparticles can improve the SAR value, but too large particles may lead to decreased colloidal stability. Therefore, researchers have developed nanoparticles with special magnetic domain structures, such as iron oxide nanorings with eddy current magnetic domains, which can have a SAR value of more than 2,000 W/g while maintaining excellent colloidal stability (Liu et al., 2015).

In practice, MHT is used as a means of *in situ* thermotherapy, and due to the excellent tissue penetration ability of AMF, MHT can deal with deep tumors in a variety of organs. MHT with magnetic iron oxide nanoparticles has been approved for the treatment of radiologically recurrent glioblastoma, and clinical trials have shown that it significantly improves the overall survival of patients (Neuwelt et al., 2015; Marchal et al., 2015). In addition, MHT can be combined with other therapies (e.g., chemotherapy, radiotherapy, and immunotherapy) to produce a synergistic effect; for example, in the treatment of localized high-risk soft tissue sarcoma, MHT combined with neoadjuvant chemotherapy resulted in a significant improvement in the progression-free survival and overall survival of patients (Issels et al., 2018).

### 4.2 Photothermal therapy (PTT)

As an emerging local tumor treatment strategy that is temporally and spatially controllable, PTT has attracted extensive attention due to its non-invasiveness, low drug resistance, and high efficiency (Yu et al., 2023). PTT uses photothermal agents to convert light energy into heat energy under near-infrared (NIR) light irradiation, thereby generating high temperatures locally in the tumor and killing cancer cells. The perfect photothermal agents should have stable NIR absorption, high photothermal conversion efficiency, and excellent biocompatibility (Liang et al., 2021).

**FIGURE 4:**
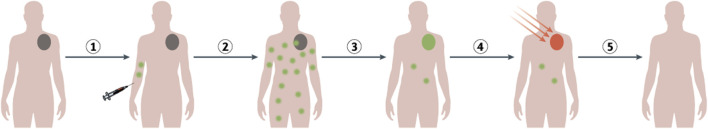
General procedure and mechanisms of action for PTT. (1) The PTT agent (photosensitizers; small green circles) is administered to the patient, typically intravenously. (2) The PTT agent is subsequently distributed around the body. (3) Accumulation of PTT agent in tumor tissues (indicated by previously grey ovals, representing the tumor, turning green) can be achieved through active and/or passive targeting strategies and optional molecular activation exploiting, for example, proteases or hypoxia in the tumor microenvironment. (4) Local application of light of a specific wavelength to the tumor tissues results in excitation of the PTT agent from a ground singlet state to an excited singlet state (indicated by red oval). (5) Tumor ablation following excitation of the PTT agent results predominantly from thermal and chemical damage, respectively (Li et al., 2020).

**FIGURE 5:**
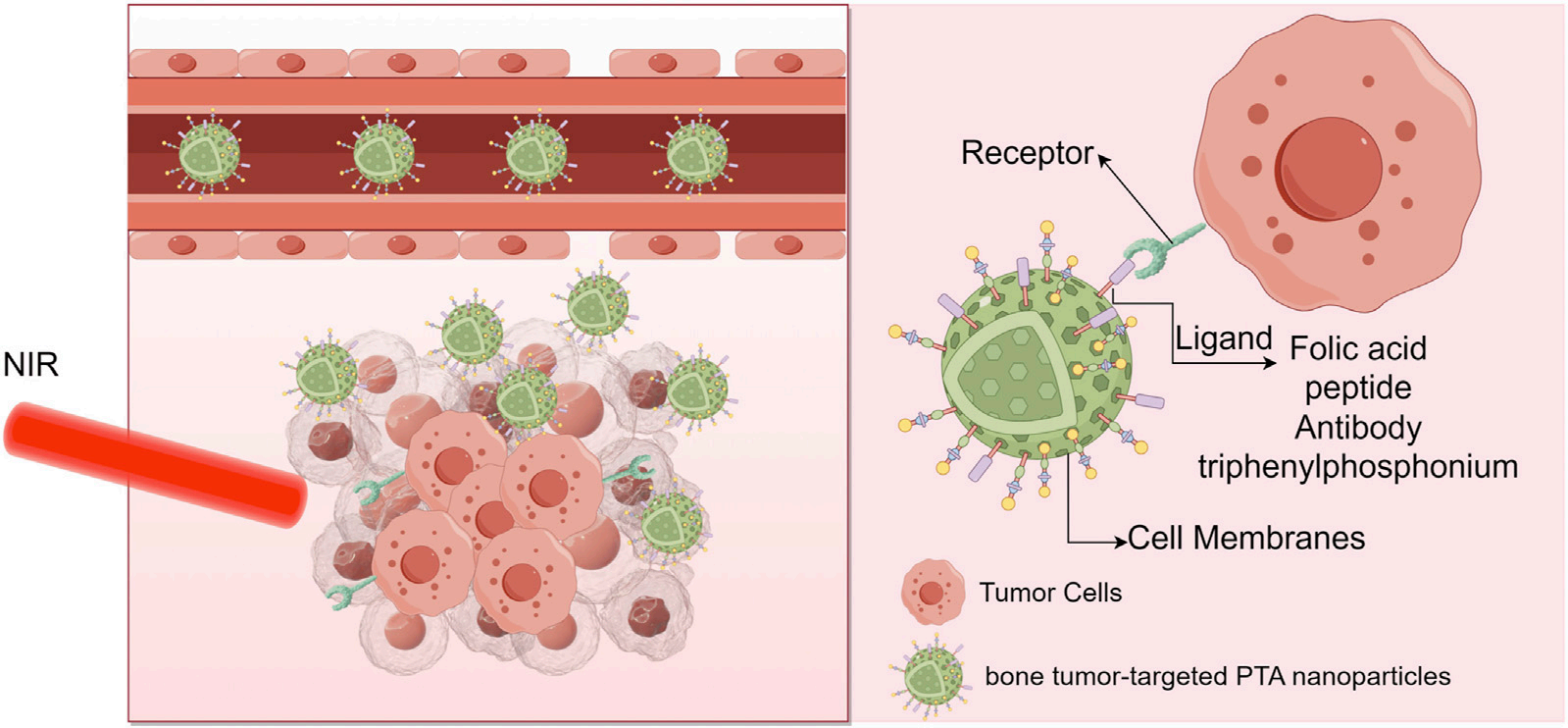
Target antitumor treatment with PTT (Xie M. et al., 2024).

In recent years, MNMs, especially those that combine magnetic and photothermal properties, have shown great potential in PTT. During the PTT process, the magnetism of these MNMs enables them to precisely locate to the tumor area under the guidance of an applied magnetic field, and then use their photothermal properties to respond to near-infrared light irradiation, producing a strong photothermal effect, causing local overheating of tumor tissue and eliminating cancer cells. Through the accumulation of MNMs in the tumor area, magnetic heating and photothermal heating can be combined for comprehensive treatment, thereby significantly improving the treatment effect. This dual-effect nanomaterial with magneto-optical properties can not only improve the efficacy, but also synchronizes diagnosis and treatment through imaging guidance by techniques such as MRI or Magnetic Particle Imaging (MPI) (Du et al., 2021). The general procedure and underlying mechanisms of PTT are illustrated in Figure 4, highlighting the delivery, accumulation, and activation of photothermal agents. Furthermore, Figure 5 presents a schematic representation of the application of PTT in targeted antitumor treatment, emphasizing the therapeutic potential of localized heating strategies.

For example, Zhang et al. developed a manganese-doped iron oxide (MnIO)-based magnetic nanoparticle that exhibited good photothermal conversion efficiency (PCE = 26.9%) under near-infrared (808 nm) irradiation and functioned as a T1-weighted MRI contrast agent, providing imaging information for tumor localization (Yu et al., 2023). Similarly, Hou’s group designed a multifunctional probe based on ${\text{Fe}}_{3}{\text{C}}_{2}$ nanoparticles, which demonstrated excellent photothermal conversion capability under the same NIR wavelength, rapidly raising the temperature from 25°C to over 42°C within 6 min to effectively induce tumor cell apoptosis (Zhang et al., 2015). In addition to their therapeutic effects, ${\text{Fe}}_{3}{\text{C}}_{2}$ nanoparticles also served as potent T2-weighted MRI contrast agents, offering intuitive imaging for tumor monitoring and treatment evaluation (Yu et al., 2016). Building upon these advances, recent developments in photothermal-responsive nanosystems, such as 980 nm laser-activated Pt (II) metallacycle nanoplatforms, have demonstrated not only high photothermal efficiency but also safety in photo-induced bacterial sterilization (Xu Y. et al., 2023). These findings further underscore the potential of integrating magnetic and photothermal functionalities into nanomaterials for a wide range of biomedical applications, including precise tumor ablation, infection control, and theranostic strategies.

### 4.3 Magnetic-targeted drug delivery (MTDD)

**FIGURE 6:**
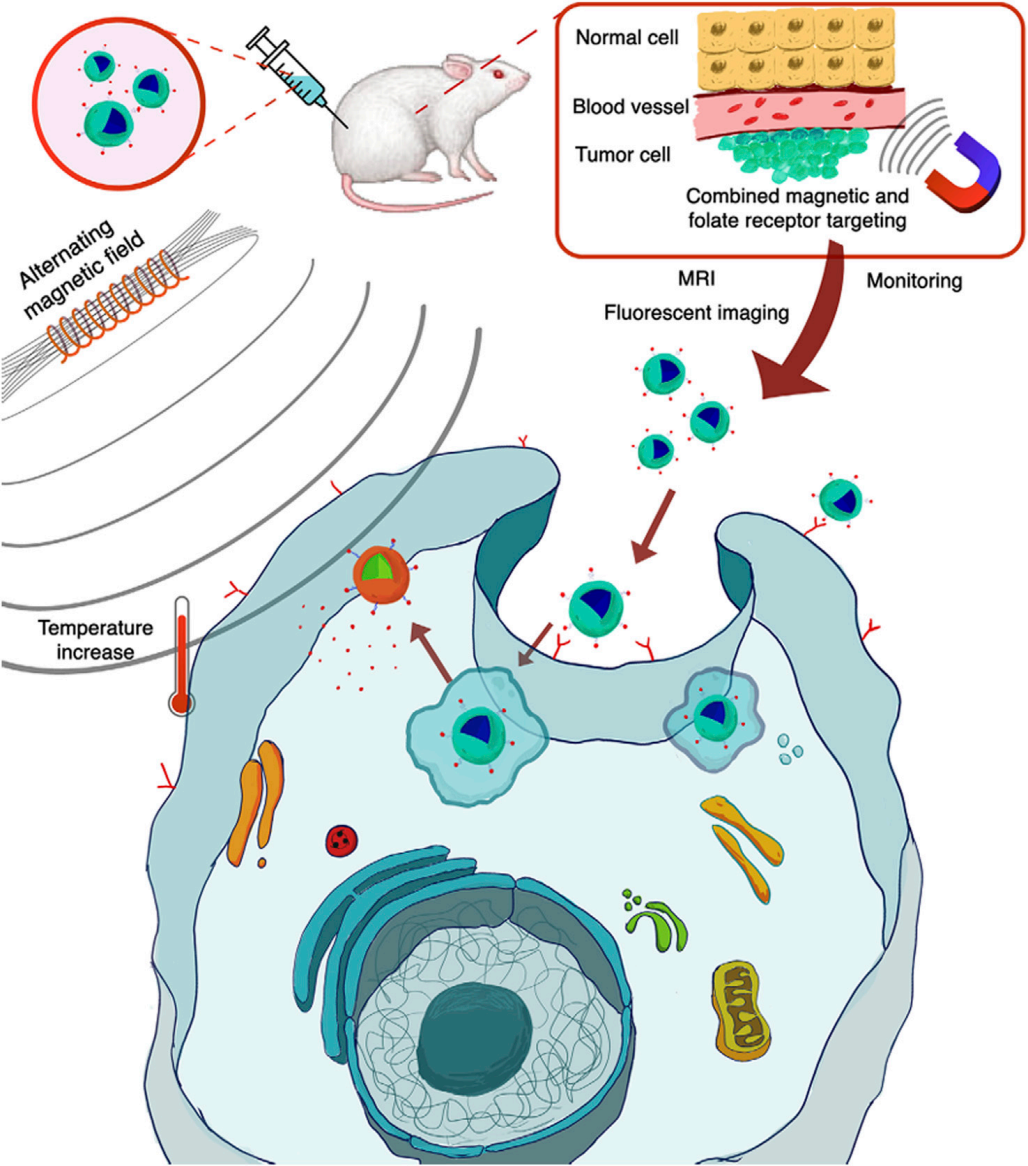
Schematic of magnetic synergistic drug delivery and magnetic hyperthermia. Magnetic nanomaterials are first guided and accumulated at the tumor site from the circulating blood under the influence of an external static magnetic field. Once localized, an AMF is applied, inducing Brownian and Néel relaxation of the nanomaterials, which generates localized heat and elevates the temperature of the tumor microenvironment. The temperature rise triggers tumor cell apoptosis or necrosis, while simultaneously promoting the release of temperature-responsive anticancer drugs loaded on the nanomaterials surfaces. This process achieves a synergistic therapeutic effect by combining magnetic hyperthermia and targeted chemotherapy. (Anik et al., 2021).

Targeted delivery of anticancer drugs remains a major challenge in current cancer therapy, and MNMs have shown significant promise in this field due to their unique biological and magnetic properties. MTDD leverages the magnetic responsiveness of MNMs, allowing them to be precisely directed to specific tissues under the guidance of an external magnetic field (Figure 6). Through surface engineering, MNMs can efficiently bind, encapsulate, and transport anticancer drugs and imaging agents, enabling controlled drug release at preselected biological sites. Moreover, their intrinsic imaging capabilities allow for real-time monitoring and guidance, enhancing the precision of drug delivery (Mok and Zhang, 2013). This strategy not only improves the bioavailability and therapeutic concentration of the drugs at the disease site but also minimizes systemic toxicity, thereby significantly enhancing overall treatment efficacy (Zhang et al., 2025).

The efficiency of MTDD is affected by a variety of factors, including the strength of the applied magnetic field, the magnetic properties of the nanomaterials, their size, shape, and surface coating, as well as being constrained by extracellular and intracellular barriers (Shapiro et al., 2015). In addition to drugs, MNMs can be loaded with other molecules such as nucleic acids, cells, proteins or antibodies. For example, superparamagnetic iron oxide nanoparticles (SPIONs) coated with paclitaxel-chitosan and folic acid-polyethylene glycol proved to be an effective strategy for targeting fibrosarcoma, inducing apoptosis in cancer cells and dramatically shrinking tumor size (Al-Obaidy et al., 2023).

## 5 Conclusion

MNMs hold great promise in the field of biomedicine, particularly in imaging and therapeutic applications. In medical imaging, MNNs serve as highly effective contrast agents for MRI, enhancing image resolution and diagnostic accuracy. Their unique magnetic properties also enable their integration into CT, PET, and multimodal imaging, providing complementary anatomical and functional insights (Yang et al., 2025a; Yang et al., 2025b). Beyond imaging, MNMs play a crucial role in MHT, where they generate localized heat under an alternating magnetic field, selectively eliminating tumor cells with minimal invasiveness. Additionally, MNMs facilitate MTDD by enabling precise external control over drug transport, thereby reducing systemic toxicity and improving therapeutic efficacy. Recent advances in nanotechnology have further expanded the application scope of nanoscale platforms in molecular diagnostics, as demonstrated by electrohydrodynamic-driven nanobox-on-mirror systems achieving high clinical accuracy in the identification of respiratory viruses (Li et al., 2024). Such innovations highlight the potential of integrating magnetic nanomaterials into next-generation theranostic strategies, seamlessly combining targeted imaging, localized therapy, and molecular-level diagnostics (Yang et al., 2023).

Despite the significant advances of magnetic nanomaterials (MNMs) in imaging and therapeutic applications, several challenges still hinder their clinical translation. Biocompatibility and long-term safety remain major concerns, as some MNMs may induce oxidative stress, release toxic metal ions, or accumulate in vital organs such as the liver and spleen (Xuan et al., 2023; Deng et al., 2024; Yang et al., 2024; Zhang Q. et al., 2024). To mitigate these risks, various strategies have been developed. Surface modification techniques, such as PEGylation, are widely employed to shield nanomaterials from immune surveillance, prolong systemic circulation, and reduce nonspecific uptake by the reticuloendothelial system. Liposomal encapsulation and polymer coatings further stabilize nanomaterials, prevent aggregation, and limit premature metal ion release. In addition, the incorporation of biodegradable materials, including polysaccharides, gelatin derivatives, and FDA-approved carriers such as ferumoxytol, has been explored to enhance metabolic clearance and minimize long-term tissue accumulation, improving biocompatibility profiles in both preclinical and clinical studies. Moreover, environmental safety is becoming an increasingly important consideration, particularly given the growing clinical and industrial deployment of nanomaterials. Designing MNMs with eco-friendly compositions and ensuring their biodegradability after use are critical for minimizing ecological risks and promoting sustainable biomedical applications. Another major hurdle lies in the precise targeting of deep-seated tumors, as the shallow penetration depth of external magnetic fields limits the efficacy of conventional magnetic targeting strategies. To address this, advanced approaches such as the use of high-gradient magnetic fields, implantable magnetic sources adjacent to tumor tissues, and dynamic magnetic navigation systems are under investigation. These methods aim to enhance targeting efficiency, improve therapeutic precision, and broaden the clinical applicability of MNM-based platforms. Collectively, addressing these biological, material, and environmental challenges will be pivotal for the successful translation of MNMs into clinical practice.

Another significant obstacle is the complexity of MNMs synthesis and large-scale production, and achieving consistent size, shape, and magnetic properties remains challenging. Addressing these issues requires further improvements in surface engineering, magnetic field control, and scalable manufacturing technologies to improve performance and promote clinical applications of precision medicine.
